# Ontogeny of CYP3A and UGT activity in preterm piglets: a translational model for drug metabolism in preterm newborns

**DOI:** 10.3389/fphar.2023.1177541

**Published:** 2023-04-13

**Authors:** Laura Buyssens, Allan Valenzuela, Sara Prims, Miriam Ayuso, Thomas Thymann, Chris Van Ginneken, Steven Van Cruchten

**Affiliations:** ^1^ Comparative Perinatal Development, Department of Veterinary Sciences, Faculty of Pharmaceutical, Biomedical and Veterinary Sciences, University of Antwerp, Wilrijk, Belgium; ^2^ Comparative Pediatrics and Nutrition, Department of Veterinary and Animal Sciences, University of Copenhagen, Frederiksberg, Denmark

**Keywords:** CYP, UGT, ontogeny, preterm, term, pig, pediatrics

## Abstract

Despite considerable progress in understanding drug metabolism in the human pediatric population, data remains scarce in preterm neonates. Improving our knowledge of the ADME properties in this vulnerable age group is of utmost importance to avoid suboptimal dosing, which may lead to adverse drug reactions. The juvenile (mini)pig is a representative model for hepatic drug metabolism in human neonates and infants, especially phase I reactions. However, the effect of prematurity on the onset of hepatic phase I and phase II enzyme activity has yet to be investigated in this animal model. Therefore, the aim of this study was to assess the ontogeny of CYP3A and UGT enzyme activity in the liver of preterm (gestational day 105–107) and term-born (gestational day 115–117) domestic piglets. In addition, the ontogeny pattern between the preterm and term group was compared to examine whether postconceptional or postnatal age affects the onset of enzyme activity. The following age groups were included: preterm postnatal day (PND) 0 (*n* = 10), PND 5 (*n* = 10), PND 11 (*n* = 8), PND 26 (*n* = 10) and term PND 0 (*n* = 10), PND 5 (*n* = 10), PND 11 (*n* = 8), PND 19 (*n* = 18) and PND 26 (*n* = 10). Liver microsomes were extracted, and the metabolism of CYP3A and UGT-specific substrates assessed enzyme activity. Preterm CYP3A activity was only detectable at PND 26, whereas term CYP3A activity showed a gradual postnatal increase from PND 11 onwards. UGT activity gradually increased between PND 0 and PND 26 in preterm and term-born piglets, albeit, being systematically lower in the preterm group. Thus, postconceptional age is suggested as the main driver affecting porcine CYP3A and UGT enzyme ontogeny. These data are a valuable step forward in the characterization of the preterm piglet as a translational model for hepatic drug metabolism in the preterm human neonate.

## 1 Introduction

In recent years, the understanding of drug metabolism in the human pediatric population has considerably progressed, but data on preterm neonates remain scarce (van den Anker and Allegaert, 2021). Since enrollment of this vulnerable pediatric subpopulation in clinical studies is restricted by several factors (e.g., difficulty in obtaining informed parental consent, limited possibilities for repeated sampling, and general lack of pediatric trials), little is known about pharmacokinetics (PK) in preterm neonates (i.e., born before 37 weeks of gestation) (Mørk et al., 2022). Understanding the ADME (absorption, distribution, metabolism, and excretion) of drugs in this population is critical, as these patients often require pharmacological treatment to survive (Mørk et al., 2022). In addition, the level of prematurity may affect drug-metabolizing enzyme (DME) ontogeny, which is insufficiently assessed in preterm neonates (Mørk et al., 2022). Gestational age (GA) (i.e., time elapsed between the first day of the last menstrual period and birth), postnatal age (PNA) (i.e., chronological age starting from the day of birth) and postmenstrual age (PMA) (i.e., sum of GA and PNA) (Engle, 2004) are known to affect the degree of enzyme maturation at birth, but the influence of these parameters on DME in preterms is poorly understood (Gow et al., 2001; Kearns et al., 2003; O'Hara et al., 2015; Mørk et al., 2022). This lack of knowledge of the biotransformation processes increases the risk of suboptimal dosing, possibly resulting in adverse drug reactions (Kearns et al., 2003; O'Hara et al., 2015; van den Anker and Allegaert, 2021; Mørk et al., 2022). The commonly used solution of allometric scaling based upon data in older children and adults has often led to incorrect dosing regimens in preterm neonates, and several examples of adverse events due to immature DME have been reported (e.g., Grey baby syndrome and gasping syndrome) (Weiss et al., 1960; Gershanik et al., 1982).

Biotransformation of xenobiotics, but also endogenous substances (e.g., bilirubin, steroid hormones, thyroid hormones, etc.), mainly occurs in the liver and is mediated by phase I and phase II reactions. Phase I enzymes aim to reduce the lipophilicity of these compounds by oxidation, reduction, and hydrolysis reactions. The resulting metabolites will be more hydrophilic and thus easier to excrete by the biliary or renal pathway. The cytochrome P450 (CYP) enzyme superfamily is one of the most important groups of phase I DME, as they are responsible for the metabolism of 70%–80% of all marketed drugs in clinical use (Zanger and Schwab, 2013). Within this superfamily, the CYP3A family is the most abundant one and metabolizes a substantial fraction of prescribed drugs in children (Ince et al., 2013). Phase II enzymes conjugate a functional group to either the parent drug or its phase I metabolites, creating more polar substances and facilitating elimination. Phase II metabolism is performed by*, inter alia*, uridine 5′-diphospho-glucuronosyltransferases (UGT), sulfotransferases, N-acetyltransferases, and glutathione S-transferases (O'Hara et al., 2015). Within this group, UGT enzymes are responsible for ∼35% of all phase II reactions (Evans and Relling, 1999). Several studies have investigated the ontogeny pattern of CYP and UGT enzymes in the neonatal, juvenile, adolescent, and adult human populations (Hines, 2013; Lu and Rosenbaum, 2014; Sadler et al., 2016; Liu et al., 2020). However, very little data is available concerning the preterm-born neonate, since liver samples are challenging to obtain from this pediatric subpopulation. Thus, the aim of this study is to investigate their ontogeny in the preterm piglet as a surrogate to better understand the biotransformation capacity in the human preterm neonate.

The preterm pig model was characterized before and found to be representative of human preterm physiology (Eiby et al., 2013). However, a direct correlation between both species based upon GA is not possible (Sangild et al., 2013), e.g., GIT maturation in the piglet is slower than in man since the development is not finished at birth and continues during the first weeks of *ex utero* life (Sangild, 2006). In general, it is assumed that 90% gestation in pigs represents a good model for the GIT system in preterm infants born at 75% gestation (Sangild, 2006). Regarding perinatal terminology, postconceptional age (PCA) (i.e., sum of GA starting from the day of conception and PNA) is used in the pig instead of PMA. Pigs have an estrous cycle (Soede et al., 2011) instead of a menstrual cycle in humans and therefore the terminology PMA is not applicable. In view of PK characteristics, multiple studies have shown considerable similarities between the juvenile population in (mini)pigs and humans (Van Peer et al., 2015; Gasthuys et al., 2016; Van Peer et al., 2017; Millecam et al., 2018). Recent research in both the conventional pig and minipig has shown similar ontogeny profiles for CYP enzyme activity and protein abundance in comparison to human neonates and infants (Van Peer et al., 2015; Van Peer et al., 2017; Millecam et al., 2018; Schelstraete et al., 2019; Buyssens et al., 2021). Despite the presence of similar ontogeny profiles, it should be taken into account that absolute enzyme activity and protein abundance levels are not necessarily the same between animal models and humans (van Groen et al., 2021). Caution is thus needed when comparing species. Concerning UGT enzymes, maturation data are limited and are primarily described in term-born neonatal and juvenile pigs (Higashi et al., 2014; Hu, 2017; Van Peer et al., 2017). Although fetal pig samples were included in some studies (Van Peer et al., 2015; Van Peer et al., 2017; Buyssens et al., 2021), the effect of preterm birth on the onset of phase I and II enzyme activity in piglets has not been investigated yet.

The main goal of this study was to assess the ontogeny of CYP3A and UGT enzyme activity in preterm and term-born domestic piglets. Enzyme activity was measured by the metabolism of enzyme-specific substrates in liver microsomes of both male and female piglets, with age groups ranging from the day of birth until postnatal day (PND) 26, the weaning age. Second, the ontogeny pattern between preterm and term animals was compared to examine whether PCA (i.e., a predetermined “biological clock”) or PNA (i.e., birth effect) (Ren et al., 2018) affects the onset of enzyme activity.

## 2 Materials and methods

### 2.1 Animals and tissue

Liver samples originating from preterm and term-born piglets (Danish Landrace x Large White x Duroc) were a kind gift from the University of Copenhagen (Andersen et al., 2016; Ren et al., 2018). All animal experiments were approved by the Danish Committee for Animal Research (license #2012-15-2934-00-193). All piglets were caesarian-section-delivered, either preterm at gestational day 105–107 (90% of gestation, 10 days before full term) or full term at gestational day 115–117 (100% of gestation). All piglets were immediately transferred to a piglet neonatal intensive care unit and reared in temperature-, moisture- and oxygen-regulated incubators. The animals were randomly allocated to a specific age group and humanely killed at the time points chosen using initial induction of anesthesia (mixture of zolazepam, tiletamine, ketamine, butorphanol, and xylazine) followed by intracardiac injection of a lethal dose of sodium pentobarbital. The following age groups were investigated: preterm PND 0 (*n* = 10), PND 5 (*n* = 10), PND 11 (*n* = 8), PND 26 (*n* = 10) and term PND 0 (*n* = 10), PND 5 (*n* = 10), PND 11 (*n* = 8), PND 19 (*n* = 18), and PND 26 (*n* = 10). The age groups of the term piglets cover the neonatal period up until infancy and correspond to the age range of 1 month to 2 years of age in humans (Gad, 2016). The preterm group was born approximately 10 days before the term group. Hence, preterm PND 11 and term PND 0 animals and preterm PND 26 and term PND 19 animals shared nearly the same PCA. Both sexes were equally represented in each age group except for preterm PND 0 (seven males and three females), term PND 5 (four males and six females), and term PND 19 (ten males and eight females). No preterm PND 19 samples were available. For the UGT activity assessment, five males and five females from the term PND 19 group were randomly selected for analysis. All term PND 19 animals were included in the CYP3A activity assessment. Isolation of the liver microsomes was performed as described by Van Peer et al. (2015). The Pierce® BCA Protein Assay Kit determined the total protein concentration of the liver microsomes, using bovine serum albumin as a standard.

### 2.2 CYP3A activity assessment

The protocol for the P450-Glo^TM^ CYP3A4 assay (V9002; Promega Corporation, WI, USA) was executed as described earlier by our research group (Van Peer et al., 2015). In brief, pig liver microsomes were incubated with Luciferin-IPA, a specific human CYP3A4 substrate. Biotransformation of this substrate by porcine CYP3A4 homologs resulted in the formation of D-Luciferin. The concentration of this metabolite was quantified based on comparing the luminescent signal of the reaction mixtures with those from a D-Luciferin (Beetle Luciferin, E1601; Promega Corporation, WI, USA) standard curve. Luminescence was measured with a Tecan Infinite M200 Pro (Tecan Group Ltd., Männedorf, Switzerland). Determination of the kinetic profile of Luciferin-IPA and optimization of the substrate concentration (1 µM), incubation time (10 min), and microsomal protein (MP) concentration (20 μg/mL) within the linear range of Luciferin-IPA were described before (Van Peer et al., 2015). CYP3A4 baculosomes (P2377; Thermo Fischer Scientific, MA, USA) and insect cell control supersomes (456200; Corning Incorporated, NY, USA) were used as positive and negative control, respectively. Both were included in each plate and treated equivalently to the pig liver microsomes. The study was performed in non-treated Nunc^TM^ F96 Microwell^TM^ white Polystyrene plates (236205; Thermo Fisher Scientific, MA, USA). Reaction velocities were calculated in units of picomoles of D-Luciferin formed per minute per milligram of microsomal protein (pmol/min/mg MP). The lower limit of detection (LLOD) and lower limit of quantification (LLOQ) were 0.97 nM and 2.3 nM, respectively. The data represent the mean value for each sample obtained in three technical replicates. The coefficient of variation (CV) for these replicates was within limits (<10%).

### 2.3 UGT activity assessment

UGT activity was assessed by a fluorescent assay (UGT Activity Assay/Ligand Screening kit, ab273331; Abcam, Cambridge, UK) based upon the following principle: two reactions are set up in parallel for each sample. In the first reaction (i.e., plus-UDPGA reaction), liver microsomes, fluorescent UGT multienzyme substrate (glucuronidated by human UGT1A1, 1A3, 1A6, 1A9, and 2B7), and UDPGA (glucuronic acid donor) are present. The second reaction (i.e., minus-UDPGA reaction) is like the first one except for UDPGA being replaced by an equal volume of UGT assay buffer. Thus, in the first reaction, the UGT substrate becomes glucuronidated over time, depending on the UGT content. The part of the substrate that remained unmodified and the substrate that was present in the minus-UDPGA reaction will produce a fluorescent signal. The decrease in fluorescent signal, measured by the difference between both reactions, is proportional to the glucuronidation activity of the sample. A standard curve with the UGT multienzyme substrate (range 0–2 nmol/well) was included in each 96-well plate. Hence, the amount of unmodified substrate during the reaction could be quantified at each time point. Human liver microsomes (HLM) (H0620; Xenotech, KS, USA) were included as a positive control. Insect cell control supersomes (456200; Corning Incorporated, Corning, NY, USA), lacking UGT activity, were included as a negative control. The positive and negative controls were treated correspondingly to the pig liver microsomes.

Determination of preterm and term-born piglet UGT activity was conducted in Greiner 96 Flat Bottom Black Polystyrene Chimney plates (655900; Greiner Bio-One, Belgium). A range of six MP concentrations for the 2X sample premix preparation (6.25, 12.5, 25, 50, 100, and 200 μg/mL) and six substrate concentrations (2.5, 5, 10, 20, 40, and 80 µM) were tested for linearity. These parameters were investigated in a pool of liver microsomes originating from four term PND 26 animals (three males and one female). This pool was created by diluting the samples to the same concentration (2,000 μg/mL) and the same volume (62.5 µL). Next, all diluted samples were pooled and mixed to a final volume of 250 µL. After this optimization step, the final MP concentration and substrate concentration were set to 50 μg/ml MP and 10 µM UGT multienzyme substrate, respectively. The final reaction volume (100 µL per well) consisted of 50 µL 2X sample premix (50 μg/ml MP and 0.25 µL Alamethicin), 2.5 µL 10X working solution (10 µM UGT multienzyme substrate), 27.5 µL UGT assay buffer and 20 µL UDPGA or an equal amount of UGT assay buffer for the minus-UDPGA samples. In the first step, the 2X sample premix was prepared by adding Alamethicin to the pig liver microsomes. This mixture was kept on ice and incubated for 15 min. The 10X working solution and UGT assay buffer were added, followed by incubation at 37°C for 5 min. To start the reaction, 5X UDPGA was added, except for the minus-UDPGA samples. Immediately after, the 96-well plate was placed in a preheated Tecan Infinite M200 Pro (Tecan Group Ltd., Männedorf, Switzerland) at 37°C, and fluorescence was measured at Ex/Em = 415/502 nm in kinetic mode for 40 min. Reaction velocities were calculated as recommended by the manufacturer and expressed in picomoles of UGT multienzyme substrate modified per minute per milligram of microsomal protein (pmol/min/mg MP). The LLOD and LLOQ were 0.003 nmol and 0.005 nmol, respectively. The data represent the mean value for each sample obtained in two technical replicates. Prior investigation showed that duplicates for this assay led to a CV within limits (<10%).

### 2.4 Mathematical and statistical analyses

Reaction velocities for both experiments were calculated with Microsoft Excel^®^ 365 (Microsoft Corporation, WA, United States). Values below the LLOQ were not considered for the statistical analysis. The reaction velocity was log-transformed for both assays to meet the assumptions of normality and homoscedasticity, which were tested by the Shapiro-Wilk and Levene’s tests, respectively. Statistical analyses and graphs were performed and created in JMP® Pro 16 (SAS Institute Inc., NC, United States). A *p*-value smaller than 0.05 was considered statistically significant. Both CYP and UGT data were fitted to a linear mixed model to assess the postnatal ontogeny profile. The 2-way interactions between age, group, and sex were included as fixed effects. Run-by-plates was added as a random effect to the model to correct for inter-run variability. A stepwise backward approach was used to simplify the starting model. Thus, all non-significant effects (*p* > 0.05) were removed. Tukey’s honest significance *post hoc* test was used to identify differences between age groups. Term PND 19 samples were not included in this analysis since no preterm counterpart was present. An unpaired Student’s t-test was performed to investigate the birth effect (PCA vs. PNA). Therefore, a comparison between (1) term PND 0 and preterm PND 11 and (2) term PND 19 and preterm PND 26 was conducted, as these subgroups shared nearly the same PCA.

## 3 Results

### 3.1 Postnatal CYP3A enzyme activity

**FIGURE 1:**
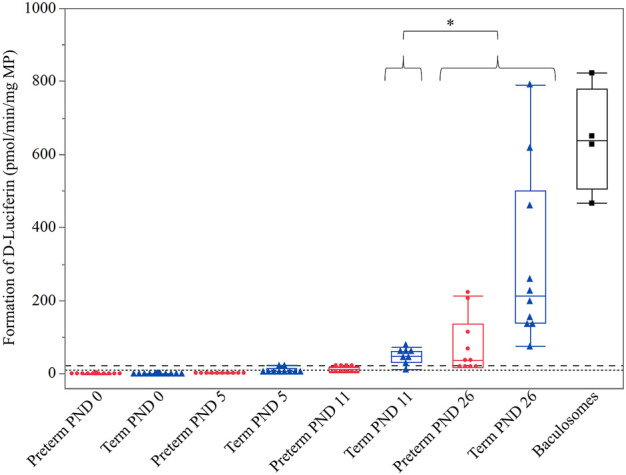
Hepatic CYP3A enzyme activity (D-Luciferin in pmol/min/mg MP) in preterm (red) and term-born (blue) piglets at different postnatal ages (PND). The mean value of three technical replicates for each sample is represented by a red dot (preterm group), a blue triangle (term group), or a black cube (baculosomes, positive control). A significantly higher CYP3A enzyme activity was detected at (term, preterm) PND 26 compared to (term) PND 11 (*: *p* = 0.0421). Irrespective of age, significantly higher CYP3A enzyme activity was detected in the term group compared to the preterm group (*p* = 0.0021; not shown in the graph for visual purposes). The upper (dashed) and lower (dotted) horizontal lines represent the LLOQ and LLOD, respectively. Values below the LLOQ were not considered for statistical analysis.

All preterm PND 0, 5, and 11 and term PND 0 and 5 values were below the LLOQ and were not included in the statistical analysis. The 2-way interactions of age*sex and group*sex were not significant (*p* = 0.8925 and *p* = 0.9631, respectively). The age*group interaction could not be assessed because only one preterm group (PND 26) was above the LLOQ. There was no significant effect of sex (*p* = 0.9818). However, a significant impact of age (*p* = 0.0402) and group (*p* = 0.0021) on CYP3A activity was detected. Accordingly, a considerably higher CYP3A enzyme activity in term-born piglets compared to preterm-born piglets was observed. On the other hand, a significantly higher CYP3A enzyme activity at term PND 26 compared to term PND 11 was detected (*p* < 0.0001) (Figure 1).

### 3.2 Effect of postconceptional age on CYP3A enzyme activity

**FIGURE 2:**
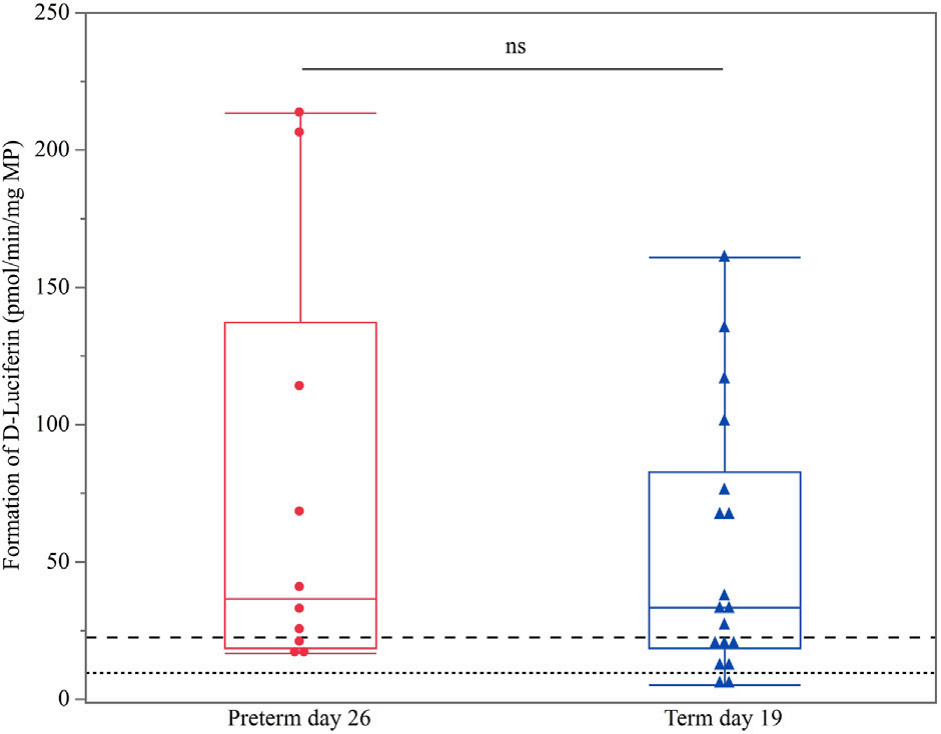
Hepatic CYP3A enzyme activity (D-luciferin in pmol/min/mg MP) in term PND 19 and preterm PND 26 piglets. These subgroups were compared as they shared nearly the same postconceptional age. The mean value of three technical replicates for each sample is represented by a blue triangle (term PND 19) or a red dot (preterm PND 26). The upper (dashed) and lower (dotted) horizontal lines represent the LLOQ and LLOD, respectively. Values below the LLOQ were not considered for statistical analysis. No statistically significant difference was observed (ns).

Term PND 0 and preterm PND 11 were below the LLOQ. As no preterm PND 19 samples were available for comparison with CYP3A enzyme activity in term PND 11 animals, only term PND 19 and preterm PND 26 values were compared. No significant difference between both groups was detected (*p* = 0.4621) (Figure 2).

### 3.3 Postnatal UGT enzyme activity

**FIGURE 3:**
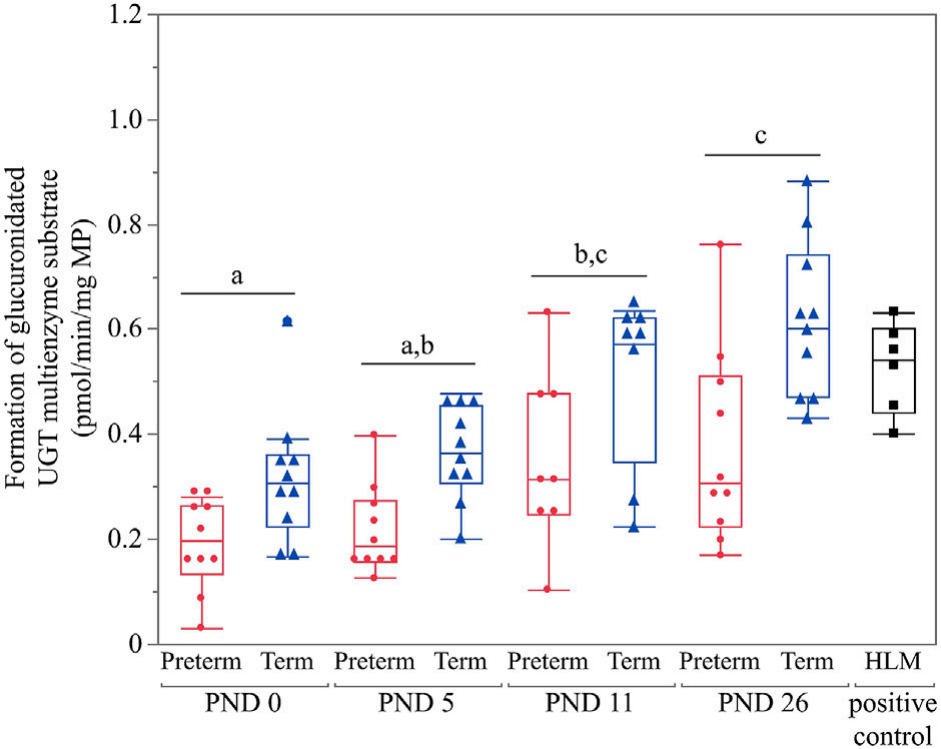
Hepatic UGT enzyme activity in preterm and term-born piglets (glucuronidated UGT multienzyme substrate in pmol/min/mg MP). The mean value of two technical replicates for each sample is represented by a red dot (preterm group), a blue triangle (term group), or a black cube (HLM, positive control). All values were above the LLOQ and thus included in the statistical analysis. Statistically significant age-related differences are indicated by characters (*p* < 0.05). Irrespective of age, significantly higher glucuronidation was detected in the term group compared to the preterm group (*p* < 0.0001).

Values for all age groups were above the LLOQ. Thus, all were included for statistical analysis. The 2-way interactions of age*group, age*sex and group*sex were not significant (*p* = 0.9433, *p* = 0.5598 and *p* = 0.1759, respectively). There was no significant effect of sex (*p* = 0.3662). However, a significant effect was detected for age (*p* < 0.0001). A gradual postnatal increase was observed from PND 0 until PND 26 for the formation of glucuronidated UGT multienzyme substrate (Figure 3). Irrespective of PNA, significantly higher glucuronidation was detected in the term group compared to the preterm group (*p* < 0.0001) (Figure 3).

### 3.4 Effect of postconceptional age on UGT enzyme activity

**FIGURE 4:**
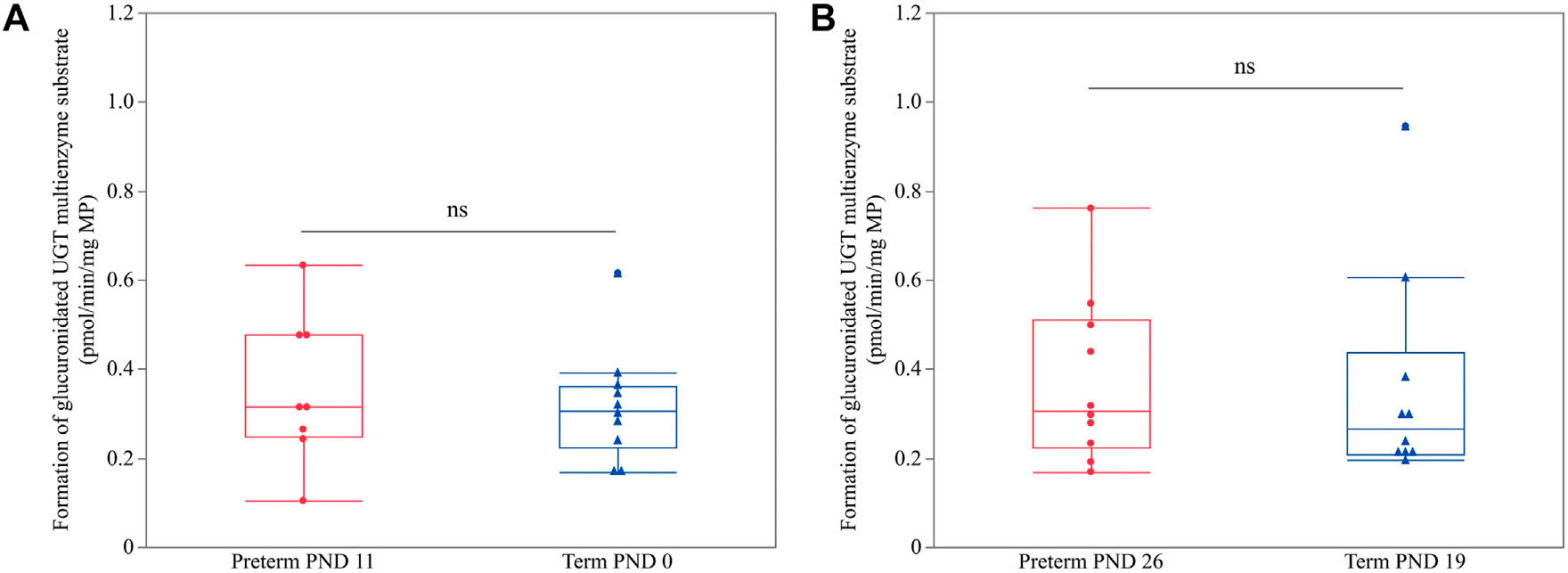
Hepatic UGT enzyme activity (glucuronidated UGT multienzyme substrate in pmol/min/mg MP) in preterm and term-born piglets. An unpaired Student’s t-test was performed to compare UGT enzyme activity between term PND 0 and preterm PND 11 **(A)** and term PND 19 and preterm PND 26 **(B)** as these subgroups shared nearly the same postconceptional age. The mean value of two technical replicates for each sample is represented by a blue triangle (term) or a red dot (preterm). No statistically significant differences were observed (ns).

No significant difference was present between term PND 0 and preterm PND 11 (*p* = 0.6154) nor between term PND 19 and preterm PND 26 (*p* = 0.7343) (Figure 4), which shared approximately the same PCA.

## 4 Discussion

The present study aimed to investigate CYP3A and UGT activity in the preterm piglet as a translational model for the biotransformation capacity in human (pre)term newborns. This is the first study to examine the ontogeny of hepatic phase I (CYP3A) and phase II (UGT) drug metabolism in the preterm-born piglet. Moreover, we wanted to address whether PCA or PNA drives enzyme activity.

### 4.1 Postnatal CYP3A enzyme activity

Pig CYP3A enzyme activity was determined in liver microsomes using the P450-Glo^TM^ CYP3A4 assay. This assay contained a highly specific human CYP3A4 substrate, Luciferin-IPA, which was shown to be metabolized by minipig CYP3A isoforms before (Van Peer et al., 2015). Since all porcine CYP3A4 orthologs (i.e., CYP3A22, CYP3A29, CYP3A39 and CYP3A46) are present in liver microsomes, no distinction was made between isoform specific activity levels in this assay. However, earlier research detected these individual isoforms at mRNA level (Nannelli et al., 2008; Puccinelli et al., 2011; Nielsen et al., 2017) and protein level (Achour et al., 2011; Millecam et al., 2018; Elmorsi et al., 2020; Buyssens et al., 2021) with the latter showing the highest abundance of CYP3A22 in developing piglets (Buyssens et al., 2021). In the preterm group, enzyme activity was only detectable at PND 26. All other age groups were below the LLOQ. In the term group, a postnatal increase in CYP3A activity was detected from PND 11 onwards. The younger age groups were below the LLOQ. These results in term-born piglets are in accordance with earlier research, in which minipig CYP3A enzyme activity was detected in term-born piglets at PND 7 with a significant increase towards PND 28 (Van Peer et al., 2015). In the same study, fetal samples (gestational days 84–86 and 108), corresponding in part to PND 0 of our preterm samples, and neonatal samples (PND 1 and 3) were included, for which values were below the LLOQ (Van Peer et al., 2015), as is the case in our study.

Considering the term CYP3A ontogeny profile, our results align with several human studies. CYP3A4 mRNA expression and enzyme activity are detected at very low levels in the fetus and start increasing at birth (Lacroix et al., 1997; de Zwart et al., 2004; Sadler et al., 2016). Later, human CYP3A4 mRNA expression, protein abundance, and enzyme activity gradually increase during postnatal life to reach 50% of adult values by 6–12 months of age (Lacroix et al., 1997; de Zwart et al., 2004; Blake et al., 2005; Hines, 2013). Although a limited number of age groups were included in this study, one can say that a similar CYP3A ontogeny pattern is observed in the pig. We focused on the pre-weaning phase, but previous research showed that CYP3A enzyme activity continues to increase gradually, after the neonatal stage, as described in humans. Millecam et al. used midazolam metabolism to measure CYP3A enzyme activity and presented a gradual postnatal increase during maturation in conventional pigs with ages ranging between PND 2 and 7 months of age (Millecam et al., 2018). This pattern was also observed in Göttingen Minipig liver microsomes incubated with midazolam. The formation of 1-OH-midazolam increased from PND 3 onwards to reach 40% of adult biotransformation capacity at PND 28 (Van Peer et al., 2017). In addition, no sex-related differences in CYP3A enzyme activity ontogeny were reported in these studies (Van Peer et al., 2017; Millecam et al., 2018), as is the case in this work. It should be considered, however, that the animals included here did not reach sexual maturity yet and that sex-related differences indeed are observed in adult pigs (Kojima and Degawa, 2016; Buyssens et al., 2021).

Next, the outcome of the term group is also in accordance with studies investigating pig CYP3A mRNA expression and protein abundance. Research has shown that neither enzyme activity nor protein abundance or mRNA expression is sufficient to predict PK characteristics as sole criterion (Heikkinen et al., 2015; Ladumor et al., 2019). Knowing the interplay of all of them is necessary to understand the underlying mechanisms of DME development. Rasmussen et al. detected significantly higher CYP3A mRNA expression in a similar breed of adult domestic pigs (Danish Landrace x Large White x Duroc) compared to fetuses (90 days of gestation) (Rasmussen et al., 2016). Even in the earlier stages of life, differential gene expression was already detected between fetal (100 days of gestation) and neonatal (PND 1) Göttingen Minipigs, with the latter having higher CYP3A mRNA expression (Hermann and Skaanild, 2011). Western blot experiments showed higher protein abundance over time in pigs, but this method often lacked sensitivity. Protein expression could be measured in adult animals, but no signal was retrieved from fetal samples (Rasmussen et al., 2016). The introduction of proteomic approaches in recent years has overcome this practical challenge. LC-MS/MS experiments in both conventional pigs and Göttingen Minipigs showed a gradual increase in CYP3A protein abundance from the fetal stage onwards, which is in line with our findings (Millecam et al., 2018; Buyssens et al., 2021).

### 4.2 Effect of postconceptional age on CYP3A enzyme activity

GA, PNA and PMA are covariates used to identify age effects (i.e., predetermined “biological clock” (GA, PMA) or birth (PNA)) on, *inter alia*, drug metabolism maturation in humans. Since PMA cannot be used in pigs, PCA is used as an alternative measure in our study. The preterm and term group showed no statistically significant difference between term PND 19 and preterm PND 26, which shared approximately the same PCA. This suggests that PCA rather than birth (PNA) affects the onset of CYP3A enzyme activity in the pig. Several human studies have shown that CYP3A activity is less mature in preterm infants compared to term newborns as measured by midazolam clearance (Jacqz-Aigrain et al., 1992; Lee et al., 1999; de Wildt et al., 2001, 2002; van Groen et al., 2019). However, research is not conclusive on whether GA, PNA or PMA affects the onset of CYP3A enzyme activity. For example, Allegaert et al. demonstrated PMA to be a better indicator than PNA during early life for *in vivo* CYP3A4 enzyme activity after a continuous tramadol infusion (Allegaert et al., 2006). Also for other CYPs, PMA is proposed to be a better predictor than PNA since maturation occurs already before birth (e.g., CYP2D6) (Anderson and Holford, 2008; Smits et al., 2012; Anderson and Holford, 2013). Next, Jacqz-Aigrain et al. determined a significant correlation between GA and midazolam clearance in preterm neonates (32.8 weeks GA) after a continuous midazolam infusion (Jacqz-Aigrain et al., 1992). The same results were obtained in a population PK study of midazolam, including (pre)term neonates born between 26 and 42 weeks of gestation (Burtin et al., 1994). Moreover, the latter study postulated that PNA did not affect midazolam kinetics (Burtin et al., 1994). Other studies, however, did not detect any correlation between midazolam clearance and age, neither for GA nor PNA (Lacroix et al., 1997; de Wildt et al., 2001, 2002). Caution is thus needed when drawing conclusions, especially when comparing species. One should acknowledge that most research assessing the relationship between CYP3A ontogeny and GA and PNA in humans is somewhat limited and was conducted 20 years ago. The current findings illustrate the importance of comprehensive PK research in preterm babies and pigs to make comparison possible.

### 4.3 Postnatal UGT enzyme activity

A UGT multienzyme substrate (specific for human UGT1A1, 1A3, 1A6, 1A9, and 2B7) was used to investigate the developmental pattern of UGT activity in preterm and term-born piglets. In contrast to the CYP experiment, UGT activity was detected for all age groups in both cohorts. In general, a gradual postnatal increase in enzyme activity during the first month of life was observed. However, a higher overall UGT activity was detected in the term group compared to the preterm group. These results are in accordance with earlier *in vitro* research in Camborough-29 pigs (Hu, 2017) and Gottingen Minipigs (Van Peer et al., 2017), which showed low UGT activity at birth, followed by an increase with age. No sex-related differences were observed, which agrees with earlier research (Van Peer et al., 2017).

Similar amino acid identities for the investigated UGT isoforms (∼70–80%) (Vaessen et al., 2017; Elmorsi et al., 2020) and substrate specificities (e.g., propofol, morphine, and ibuprofen) between (mini)pigs and humans have been reported (Matal et al., 2008; Liang et al., 2011; Higashi et al., 2014; Millecam et al., 2019), but the comparison between ontogeny profiles has not been described until now. In humans, it is generally stated that hepatic UGT enzyme activity is detected from the late second or early third trimester of gestation, followed by a boost after birth reaching full capacity between 3 months (e.g., UGT1A1) and 20 years of age (e.g., UGT1A6) (Onishi et al., 1979; Kawade and Onishi, 1981; Strassburg et al., 2002; Miyagi and Collier, 2007, 2011; Krekels et al., 2012; Rowland et al., 2013; Badée et al., 2019). As a UGT multienzyme substrate was used in this study, a direct comparison between human and pig UGT isoforms is not possible. Though, it can be confirmed that UGT enzyme activity rises after birth during the first month of life in the pig, as is the case in human neonates.

### 4.4 Effect of postconceptional age on UGT enzyme activity

Several studies in preterm and term-born human neonates indicated that the development of UGT activity occurs irrespective of GA (Onishi et al., 1979; Kawade and Onishi, 1981; Burchell et al., 1989). In fact, a positive correlation between UGT activity (e.g., UGT1A3, 1A6, 1A9, and 2B7) and PNA was described before for several substrates (e.g., morphine, propofol, paracetamol, ibuprofen, etc.) (Strassburg et al., 2002; Bouwmeester et al., 2004; Zaya et al., 2006; Miyagi et al., 2012; Smits et al., 2012; Neumann et al., 2016; Anderson and Holford, 2018; Badée et al., 2019). These observations contrast with what is detected in the present study: the lack of significant differences between equivalent PCA, namely (1) term PND 0 and preterm PND 11 and (2) term PND 19 and preterm PND 26, suggests that PCA and not PNA affects UGT development in the pig. It is thus tempting to assume that UGT development is regulated differently in humans and pigs. However, as we used a UGT multienzyme substrate in our study, studies with UGT-specific isoform substrates should be performed to either confirm or reject this assumption. Further characterization of UGT development in the preterm pig model is evidently needed.

## 5 Conclusion

This study was the first to investigate hepatic CYP3A and UGT enzyme activity in preterm and term-born piglets. CYP3A enzyme activity was only detected in preterm PND 26 piglets, while a gradual increase was observed in term-born piglets from PND 11 onwards. UGT enzyme activity showed a significant increase between PND 0 and PND 26 in both preterm and term-born piglets. The activity of both studied enzymes was lower in the preterm piglets, irrespective of PNA, suggesting that PCA is affecting CYP3A and UGT enzyme ontogeny in the pig. Our data are a valuable step forward in the characterization of the preterm piglet as a translational model for hepatic drug metabolism in the preterm human neonate.

## Data Availability

The raw data supporting the conclusion of this article will be made available by the authors, without undue reservation.

## Abbreviations

ADME, Administration, distribution, metabolism and excretion; CV, Coefficient of variation; CYP, Cytochrome P450; DME, Drug metabolizing enzymes; GA, Gestational age; GIT, Gastrointestinal tract; HLM, Human liver microsomes; LC-MS/MS, Liquid chromatography tandem mass spectrometry; LLOD, Lower limit of detection; LLOQ, Lower limit of quantification; MP, Microsomal protein; PCA, Postconceptional age; PK, Pharmacokinetics; PMA, Postmenstrual age; PNA, Postnatal age; PND, Postnatal day; UGT, uridine 5′-diphospho-glucuronosyltransferase.
